# Hypothyroidism in the “Arnolfini Portrait” (1434) by Jan Van Eyck (1390–1441)

**DOI:** 10.1007/s40618-017-0751-6

**Published:** 2017-09-09

**Authors:** H. Ashrafian

**Affiliations:** The Department of Surgery and Cancer, Imperial College London, 10th Floor Queen Elizabeth the Queen Mother (QEQM) Building, St Mary’s Hospital, Praed Street, London, W2 1NY UK

**Keywords:** Hypothyroidism, History, Ptosis, Hertoghe

## Abstract

**Background:**

The Arnolfini portrait painted by Jan van Eyck in 1434 remains one of the most puzzling yet alluring paintings of prerenaissance western art.

**Purpose:**

The painting is renowned for its exactitude in brush strokes, textures and the distinctive morphology of the main character Arnolfini. The nature of these requires pathological consideration.

**Methods:**

Diagnostic and pathological analysis of the painting.

**Results:**

A number of pathological abnormalities are noted in the face of the main character including loss of outer third of the eyebrow (Sign of Hertoghe), bilateral ptosis and melasma of the forehead. These together support a diagnosis of hypothyroidism.

**Conclusions:**

This novel diagnosis offers an additional perspective to this enigmatic portrait, and can add to the comprehension of the method, origin and pathological associations of this prominent painting from a genius artist.

The Arnolfini portrait (Fig. 1a) remains one of the most puzzling yet alluring paintings of pre-renaissance western art. Painted by Jan van Eyck in 1434, it represents a prominent Italian cloth merchant (most probably Giovanni di Nicolao Arnolfini) who was based in Bruges with his wife.

**Fig. 1:**
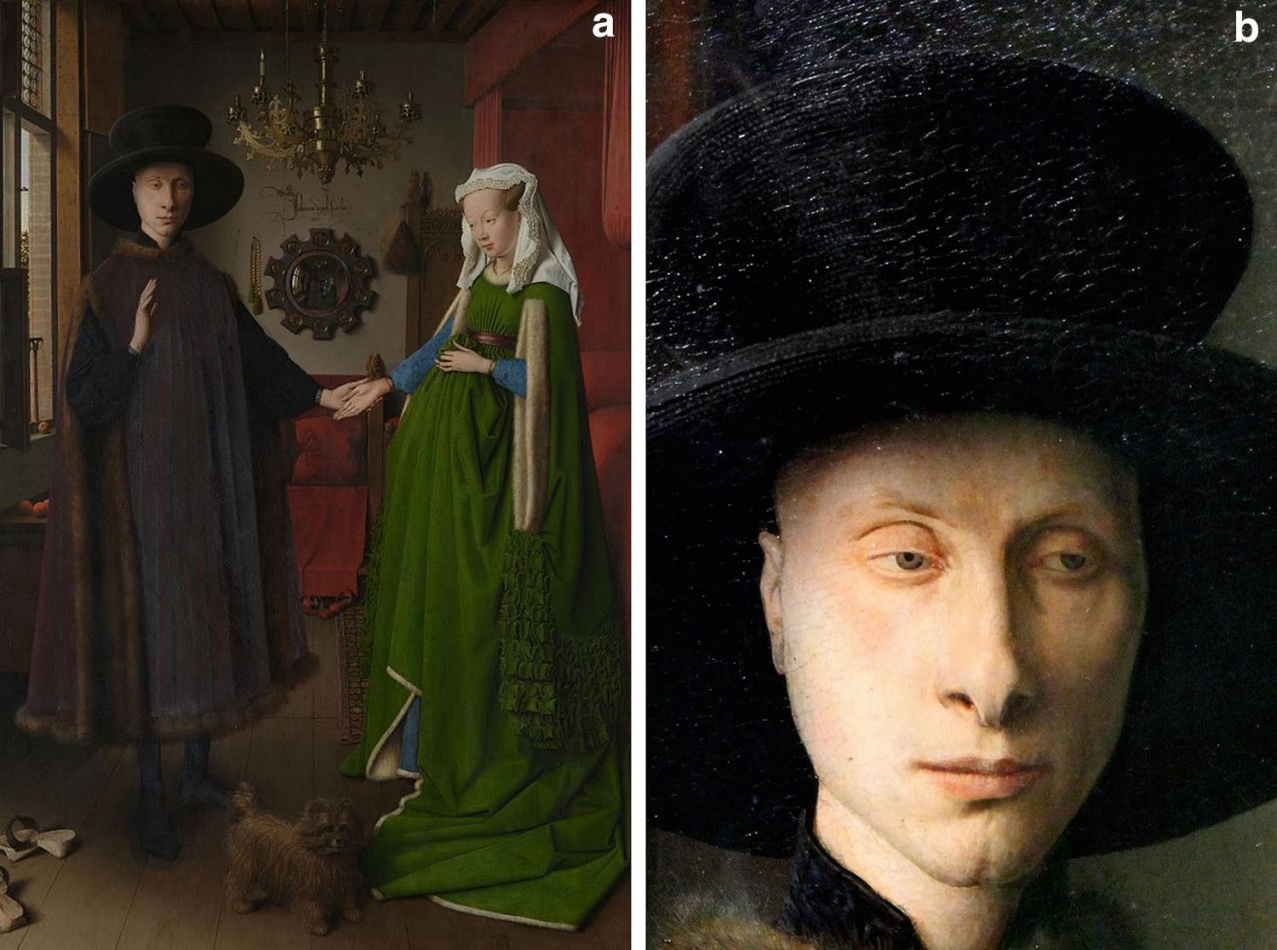
Arnolfini portrait, Jan Van Eyck (1434), **a** complete portrait; **b** close-up of the face of Arnolfini© The National Gallery, London

The painting is renowned for its exactitude in brush strokes, textures, and the distinctive morphology of the main characters, specifically Giovanni di Nicolao Arnolfini himself. A little progress, however, has been made in understanding some core features in the painting. These include: (a) the abnormal facial morphology of Arnolfini and (b) the reason why he is wearing lavish and heavy insulating clothes (particularly as the painting is likely in the summer as the trees outside are bearing summer fruits).

Assessing Arnolfini’s features (Fig. 1b), the following can be discerned: (a) he has a positive Hertoghe’s sign (loss of outer third of the eyebrow), (b) bilateral ptosis is present, and (c) there is melasma of the forehead. Together, these support a diagnosis of hypothyroidism. Finally, although we do not get a view of his neck, the fact that he is wearing warm clothes and a large hat in summer time also suggests (d) cold intolerance, further supporting a diagnosis of hypothyroidism.

Of note, Hertoghe’s sign is also known as Queen Anne’s sign. Whilst the exact Queen Anne associated with this eyebrow sign remains disputed, those considered as possible candidates (Anne of Denmark, Anne of France, Anne of Brittany, Anne of Austria, Anne Boleyn, and Anne of Cleves) all lived after this painting, rendering this portrait the first depiction of this sign.

An underlying diagnosis of hypothyroidism in the Arnolfini portrait offers an additional perspective to this enigmatic portrait, and can add to the comprehension the method, origin, and pathological associations of this prominent painting from a genius artist.

